# Preparation and characterization of chemically bonded argon–boroxol ring cation complexes†
†Electronic supplementary information (ESI) available: Mass spectra and IR photodissociation spectra for ^10^B-enriched species, theoretical geometry optimization and frequency simulations for isomers, and detailed bonding patterns of Ar–B interaction with QTAIM and EDA-NOCV methods. See DOI: 10.1039/c7sc02472j



**DOI:** 10.1039/c7sc02472j

**Published:** 2017-07-17

**Authors:** Jiaye Jin, Wei Li, Yuhong Liu, Guanjun Wang, Mingfei Zhou

**Affiliations:** a Department of Chemistry , Collaborative Innovation Center of Chemistry for Energy Materials , Shanghai Key Laboratory of Molecular Catalysis and Innovative Materials , Fudan University , Shanghai 200433 , China . Email: mfzhou@fudan.edu.cn

## Abstract

The cation complexes [ArB_3_O_4_]^+^, [ArB_3_O_5_]^+^, [ArB_4_O_6_]^+^ and [ArB_5_O_7_]^+^ were prepared *via* a laser vaporization supersonic ion source in the gas phase. Their vibrational spectra were measured *via* mass-selected infrared photodissociation spectroscopy. Spectroscopy combined with quantum chemical calculations revealed that the [ArB_3_O_5_]^+^, [ArB_4_O_6_]^+^ and [ArB_5_O_7_]^+^ cation complexes have planar structures each involving an aromatic boroxol ring and an argon–boron covalent bond. In contrast, the [ArB_3_O_4_]^+^ cation is characterized to be a weakly bound complex with a B_3_O_4_^+^ chain structure.

## Abstract 2

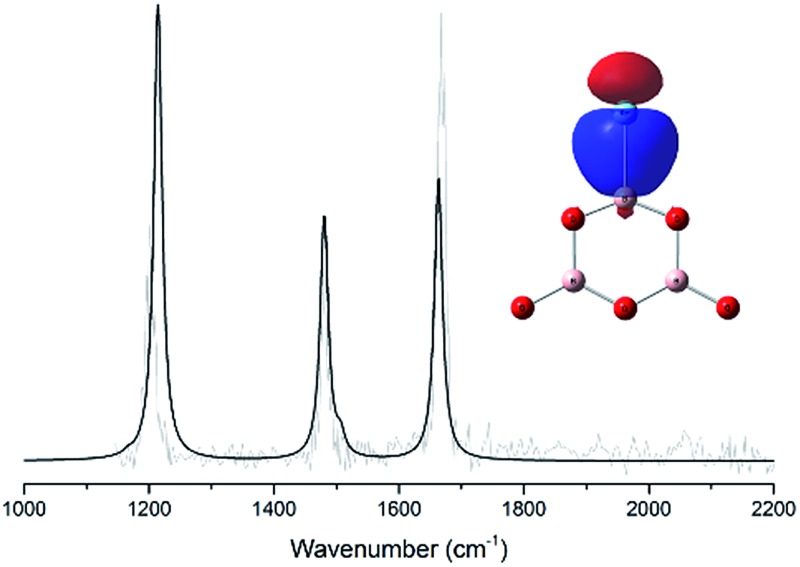
Infrared spectroscopy combined with quantum chemical calculations indicates that the [ArB_3_O_5_]^+^, [ArB_4_O_6_]^+^ and [ArB_5_O_7_]^+^ cation complexes each involve an aromatic boroxol ring and an argon–boron covalent σ bond.

## Introduction

The first synthesis of the stable xenon-containing compounds XePtF_6_, XeF_4_ and XeF_2_^1^ inspired great interest in searching for new noble gas compounds to improve our understanding of noble gas chemistry. Since then, many chemically bound compounds containing heavier noble gas atoms have been prepared and structurally characterized, most of them being xenon compounds.^2–5^ The lighter rare gas argon is even more chemically inert than the heavier noble gas atoms due to its stable valence shell. Although a number of theoretical studies indicate that chemical bonding between argon and other atoms is possible to form some metastable argon-containing compounds,^6^ HArF is presently the only experimentally known neutral molecule containing a chemically bound argon atom that is stable in a low temperature argon matrix.^7^ Theoretical calculations indicated that the H–Ar bond in HArF is covalent, whereas the Ar–F bond is mainly electrostatic.^8^ The bare ArH^+^ ion has been detected in cosmic dust associated with the Crab Nebula supernova, and is the first noble-gas molecule detected in outer space.^9^ Argon can act as a Lewis base that is able to coordinate to positively charged metal centers. Complexes containing argon such as ArBeX (X = O, S, or CO_3_),^10,11^ ArW(CO)_5_,^12^ ArMX (M = Cu, Ag, Au, *etc.*, and X = F, Cl, Br or O),^13–15^ CUO(Ar)^16^ and Ar-mixed gold–silver trimer cations^17^ have been experimentally observed in the gas phase or in low-temperature matrices. Most of the Ar–M interactions in these complexes can be regarded as Lewis acid–base interactions with quite large binding energies (about 5–10 kcal mol^–1^), but the electron density distribution analyses indicate that they have some dative bonding property instead of normal dative or electron-sharing covalent bonds.^18^

Significant efforts have also been made in searching for chemical bonding between argon and more challenging elements such as boron. Argon–boron containing compounds including FArBO, FArBF_2_ and FArBN^–^ were theoretically studied, and were predicted to be thermally metastable species with covalent Ar–B bonding.^19^ Argon coordination complexes including ArBBAr, ArB^+^, ArBF_2_^+^ and B_3_Ar_3_^+^ have also been studied computationally,^20–23^ and some of them exhibit remarkably strong Ar–B bonding.^22,23^ Only the BAr^+^ and ArBF_2_^+^ cations as well as the metastable ArBF_*n*_^2+^ dications have been experimentally detected using mass spectrometry in the gas phase.^24,25^ Here we report the experimental preparation of a group of boron oxide–argon cation complexes in the gas phase. Mass-selected infrared photodissociation spectroscopy and high-level quantum chemical calculation studies reveal strong argon–boron chemical bonding in these cation complexes featuring an aromatic boroxol ring.

## Experimental and theoretical methods

The experiment was performed using a collinear tandem time-of-flight mass spectrometer equipped with a pulsed laser vaporization/supersonic expansion cluster ion source that was described in detail previously.^26^ The cation clusters were produced by pulsed laser (532 nm, Spectral Physics, 10 Hz repetition rate) ablation of a target made of ^10^B-depleted or ^10^B-enriched powders (Alfa Aesar, 90–97%). The clusters were entrained by a helium carrier gas seeded with 0.1–1.0% O_2_ and 5–10% argon at a backing pressure of 0.6–1.2 MPa and underwent a supersonic expansion (General Valve Series 9) to form a collimated and cold molecular beam. The composition and cooling of the cation clusters were controlled by the time delay between the carrier gas pulse and the ablation laser. The cation clusters were extracted and analysed with a time-of-flight mass spectrometer (TOFMS). The ions of interest were mass selected, decelerated and then subjected to infrared photodissociation. The fragment ions together with the undissociated parent ions were reaccelerated and detected by a second collinear TOFMS. IR spectra were recorded by monitoring the relative yield of fragment ions as a function of the photodissociation IR laser wavelength. The infrared dissociation laser was generated by an OPO/OPA system (Laser Vision) pumped by a Continuum Surelite EX Nd:YAG laser, producing tunable infrared light with energies of 0.4–1.0 mJ per pulse in the wavelength range of 1150–2300 cm^–1^. The spectra were recorded by scanning the dissociation laser in steps of 2 cm^–1^ and averaging over 400 laser shots at each step.

The search for the global minimum structure of the [ArB_3_O_4_]^+^, [ArB_3_O_5_]^+^, [ArB_4_O_6_]^+^ and [ArB_5_O_7_]^+^ cation complexes and the bare [B_*x*_O_*y*_]^+^ cation clusters was performed at the B3LYP-D3/aug-cc-pVTZ^27–29^ level of theory using the Gaussian 09 program^30^ with the empirical dispersion correction added to increase the accuracy on the long-range force.^31^ The lowest energy structures were then re-optimized at the CCSD(T)/cc-pVTZ^32^ level of theory using the MOLPRO 2010 program.^33^

Chemical bonding analyses were performed by the adaptive natural density partitioning (AdNDP) method,^34^ quantum theory of atoms in molecules (QTAIM)^35^ and energy decomposition analysis with natural orbitals of chemical valence (EDA-NOCV).^36^ AdNDP is a theoretical tool for obtaining patterns of chemical bonding based on the concept of the electron pair as the main element of chemical bonding models. It achieves seamless description of systems featuring both localized and delocalized bonding without invoking the concept of resonance.^34^ The AdNDP analyses were performed using the Multiwfn program.^37^ The wave function files generated from CCSD(T) calculations were employed to analyse the topology of the electron density with the AIMALL package.^38^ The critical points (atom, bond and ring), bond paths, and Laplace distribution of density provided a comprehensive view of the electron density between the boron and argon. The EDA-NOCV bonding was analysed at the BP86/TZ2P^39–41^ level with the geometries optimized at the CCSD(T) level using the ADF2014.10 program package.^42^ The EDA-NOCV bonding analysis focuses on the instantaneous interaction energy, Δ*E*_int_, of a bond A–B between two fragments, A and B, in the particular electronic reference state and in the frozen geometry of AB. This Δ*E*_int_ is divided into three main components: the quasiclassical electrostatic interaction energy (Δ*E*_elstat_), the Pauli repulsion energy (Δ*E*_Pauli_) and the orbital interaction (Δ*E*_orb_). The Δ*E*_orb_ accounts for the charge transfer and polarization effects, and is decomposed into contributions from each irreducible representation of the point group for the system.

## Results and discussion

**Fig. 1:**
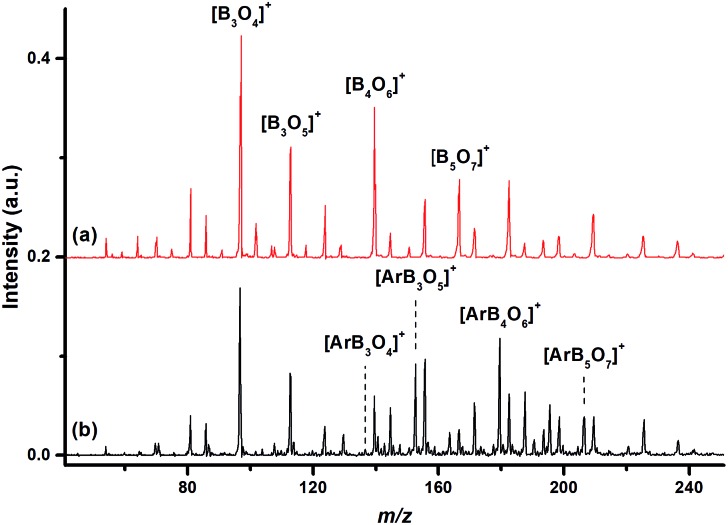
Mass spectra of boron oxide cation clusters in the *m*/*z* range of 40–250 produced by pulsed laser vaporization of a ^10^B-depleted boron target in the expansion of (a) helium seeded with 1% O_2_ (red line), and (b) helium seeded with 10% argon and 1% O_2_ (black line).

The mass spectrum using a ^10^B-depleted boron target in expansions of helium gas seeded with oxygen in the *m*/*z* range of 40–250 is shown in Fig. 1(a). The peaks due to ^11^B_3_O_4_^+^, ^11^B_3_O_5_^+^, ^11^B_4_O_6_^+^ and ^11^B_5_O_7_^+^ are the most intense peaks in the mass spectrum, in agreement with that reported in the literature.^43^ Fig. 1(b) shows the mass spectrum under the same experimental conditions but with an additional 10% argon seeded in the expansion gas. The ^11^B_3_O_4_^+^ ion remained the most intense peak, but the relative intensities of the ^11^B_3_O_5_^+^, ^11^B_4_O_6_^+^ and ^11^B_5_O_7_^+^ peaks were highly reduced. The [Ar^11^B_3_O_5_]^+^, [Ar^11^B_4_O_6_]^+^ and [Ar^11^B_5_O_7_]^+^ mass peaks were produced with quite high abundance. Similar spectra were produced using a ^10^B-enriched target (Fig. S1 of ESI†).

**Fig. 2:**
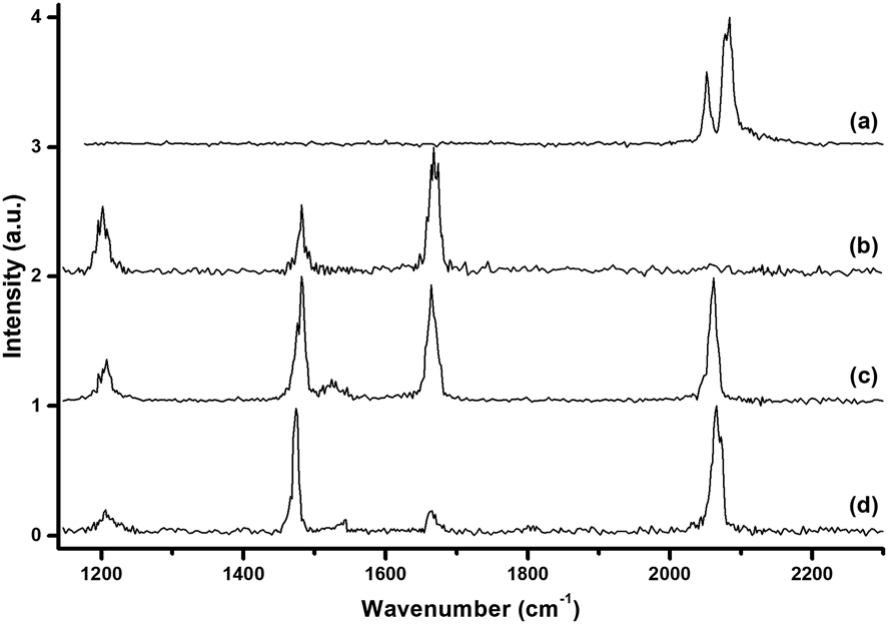
The infrared photodissociation spectra of mass-selected cation clusters in the 1150–2300 cm^–1^ region measured by monitoring the argon photodissociation channel. (a) [Ar^11^B_3_O_4_]^+^, (b) [Ar^11^B_3_O_5_]^+^, (c) [Ar^11^B_4_O_6_]^+^, and (d) [Ar^11^B_5_O_7_]^+^.

The [Ar^11^B_3_O_4_]^+^, [Ar^11^B_3_O_5_]^+^, [Ar^11^B_4_O_6_]^+^ and [Ar^11^B_5_O_7_]^+^ cations were each mass-selected and subjected to infrared photodissociation. It was found that the [Ar^11^B_3_O_4_]^+^ cation dissociated *via* losing the argon atom very efficiently and reached dissociation saturation at a quite low IR laser energy (about 0.5 mJ per pulse, see Fig. S2†). In contrast, the other ions dissociated with very low efficiency with the focused IR laser beam. As shown in Fig. S2,† the dissociation efficiency only reached about 5–6% at the highest available laser energy (1.0 mJ per pulse) at 2062 cm^–1^ for [Ar^11^B_4_O_6_]^+^. These observations indicate that [Ar^11^B_3_O_4_]^+^ is a weakly bound argon-tagged complex,^44^ while the [Ar^11^B_3_O_5_]^+^, [Ar^11^B_4_O_6_]^+^ and [Ar^11^B_5_O_7_]^+^ cations are more strongly bound. The infrared photodissociation spectra of these ions in the 1150–2300 cm^–1^ frequency region are shown in Fig. 2. The [Ar^11^B_3_O_4_]^+^ cation exhibited only two bands in the 2050–2150 cm^–1^ region, whereas three bands in the 1200–1700 cm^–1^ region were observed for the [Ar^11^B_3_O_5_]^+^ cation. The spectra of [Ar^11^B_4_O_6_]^+^ and [Ar^11^B_5_O_7_]^+^ in the 1200–1700 cm^–1^ region are quite similar to that of [Ar^11^B_3_O_5_]^+^, suggesting that [Ar^11^B_3_O_5_]^+^ may be the core structure of [Ar^11^B_4_O_6_]^+^ and [Ar^11^B_5_O_7_]^+^. All of the bands were blue-shifted in the experiments using the ^10^B-enriched target (see Fig. S3†). The observed frequency shifts (Table 1) with ^10^B/^11^B isotopic ratios in the range of 1.023–1.036 indicate that all of these bands are BO stretching vibrations. The bands above 2050 cm^–1^ are assigned as stretching vibrations of terminally bonded B≡O fragments; the bands in the range of 1400–1700 cm^–1^ can be attributed to the vibrations of the aggregated boroxol ring, and the bands near 1200 cm^–1^ fall into the region of the stretching vibrations of B–O single bonds.^45^

**Table 1:** Experimental and calculated (B3LYP-D3/aug-cc-pVTZ, unscaled) infrared frequencies (in cm^–1^, the calculated IR intensities are listed in parentheses in km mol^–1^) and isotopic shifts *Δ* (cm^–1^) of [ArB_3_O_4_]^+^, [ArB_3_O_5_]^+^, [ArB_4_O_6_]^+^ and [ArB_5_O_7_]^+^

	Exptl.			Calcd		
	^11^B	^10^B	*Δ*	^11^B	^10^B	*Δ*
[ArB_3_O_4_]^+^				2144.8(6)	2217.1(6)	+72.8
	2052	2120	+68	2123.3(1868)	2193(1770)	+69.9
	2084	2138	+54	2086.9(1135)	2162.1(1135)	+75.2
[ArB_3_O_5_]^+^	1668	1722	+54	1681.7(684)	1740.4(707)	+58.7
				1523.6(46)	1582.3(47)	+58.7
	1482	1536	+54	1496.9(595)	1548.4(663)	+51.5
	1202	1230	+28	1227.9(1119)	1259.9(1186)	+32.0
[ArB_4_O_6_]^+^	2062	2132	+70	2108.8(534)	2181.6(596)	+72.8
	1664	1724	+60	1683.2(581)	1742.1(604)	+58.9
	1524	1574	+50	1548.0(555)	1601.5(495)	+53.5
	1482	1528	+46	1495.9(858)	1546.7(1029)	+50.8
	1208	1240	+32	1234.5(1296)	1269.2(1350)	+34.7
[ArB_5_O_7_]^+^	2066	2130	+64	2114.4(808)	2187.4(862)	+73.0
				2104.9(236)	2177.7(250)	+72.8
	1666	1722	+56	1684.5(521)	1743.5(544)	+59.0
	1544	1598	+54	1570.2(386)	1623.3(401)	+53.1
	1478	1522	+44	1501.6(1824)	1547.6(1968)	+46.0
	1206	1244	+38	1242.0(1478)	1279.9(1485)	+37.9

**Fig. 3:**
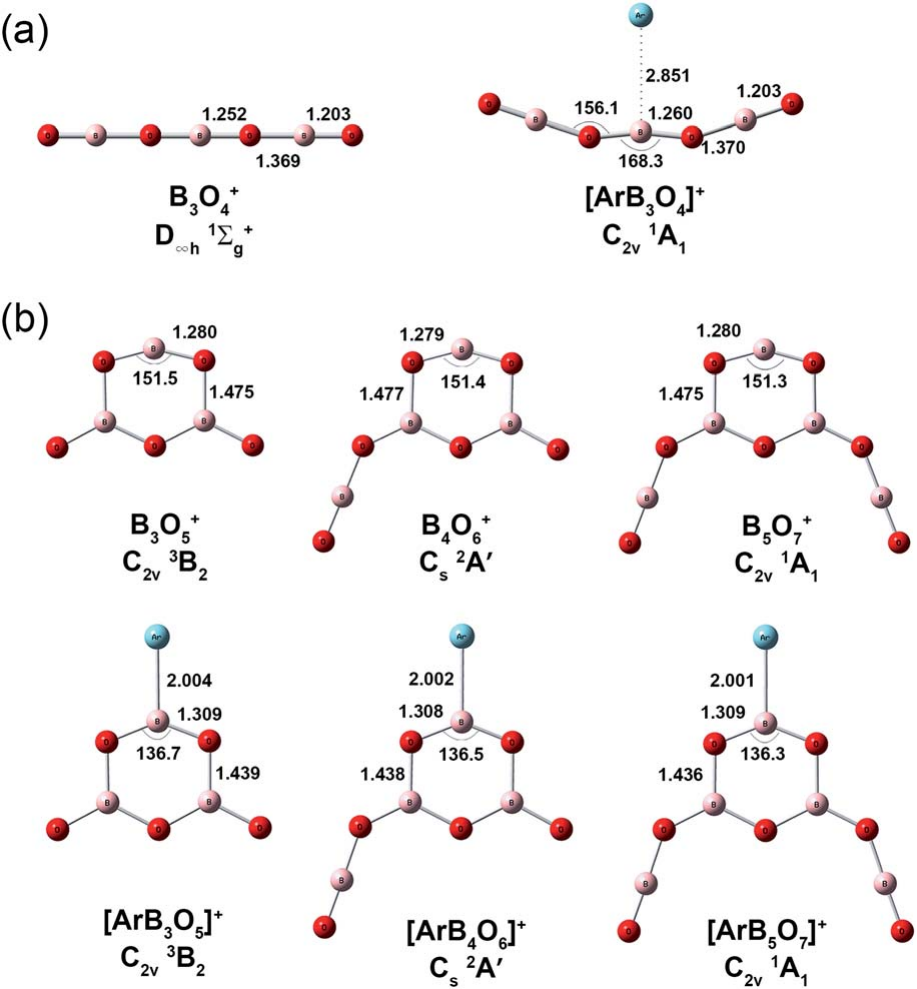
Calculated geometries of the most stable structures of the [ArB_3_O_4_]^+^, [ArB_3_O_5_]^+^, [ArB_4_O_6_]^+^ and [ArB_5_O_7_]^+^ cation complexes and the bare [B_3_O_4_]^+^, [B_3_O_5_]^+^, [B_4_O_6_]^+^ and [B_5_O_7_]^+^ cations at the CCSD(T)/cc-pVTZ level of theory. The bond lengths are in angstroms and the bond angles are in degrees.

Quantum chemical calculations were carried out to investigate the geometric and electronic structures and the vibrational frequencies of the cation complexes. Fig. 3 shows the optimized geometries of the most stable structures of [ArB_3_O_4_]^+^, [ArB_3_O_5_]^+^, [ArB_4_O_6_]^+^ and [ArB_5_O_7_]^+^ at the CCSD(T)/cc-pVTZ level of theory. Geometric optimizations were also performed on various other possible structures at the B3LYP-D3/aug-cc-pVTZ level, and the results are shown in Fig. S4–S6.† The lowest-lying structure of [ArB_3_O_4_]^+^ was identified as having a ^1^A_1_ ground state with planar *C*_2v_ symmetry involving a B_3_O_4_ chain and a weakly tagging argon. The most stable structure of [ArB_3_O_5_]^+^ has a ^3^B_2_ ground state with planar *C*_2v_ symmetry involving a boroxol ring. The argon atom is bound to one boron center of the boroxol ring, while the other two boron centers of the boroxol ring are each bonded by a monovalent O radical. The most stable structure of [ArB_4_O_6_]^+^ can be regarded as being formed *via* replacing an O radical of [ArB_3_O_5_]^+^ with an OBO fragment, resulting in a ^2^A′ ground state with planar *C*_s_ symmetry. The most stable structure of [ArB_5_O_7_]^+^ has a closed-shell singlet ground state with both O radicals of [ArB_3_O_5_]^+^ replaced by OBO. It should be noted that there are conformational isomers for both [ArB_4_O_6_]^+^ and [ArB_5_O_7_]^+^ (see Fig. S5,† structures 2A and B for [ArB_4_O_6_]^+^, and 3A–C for [ArB_5_O_7_]^+^). These isomers are isoenergetic with similar infrared spectra (Fig. S9 and S10†). Thus, the experimentally observed cations may be due to mixtures of these conformational isomers. As shown in Table 1 and Fig. S7–S10,† the calculated vibrational frequencies and boron isotopic shifts of the most stable structures for these cations were in quite good agreement with the experimental values.

The Ar–B bond distances were predicted to be 2.004, 2.002 and 2.001 Å for the [ArB_3_O_5_]^+^, [ArB_4_O_6_]^+^ and [ArB_5_O_7_]^+^ cation complexes, respectively, at the CCSD(T)/cc-pVTZ level. These Ar–B bond distances are much shorter than the sum of the van der Waals radii of argon and boron (Ar + B = 1.88 + 2.08 = 3.96 Å),^46^ and are close to the sum of their single-bond covalent radii (Ar + B = 1.81 Å).^47^ This suggests strong interaction between argon and boron in these cation complexes. The Wiberg bond orders (Table 2) were predicted to be 0.543, 0.545 and 0.547, respectively, slightly smaller than that of the H–Ar bond in HArF.^18*a*^ The bond dissociation energies (Table 2) of the argon atom were 16.2, 16.3 and 16.4 kcal mol^–1^ for [ArB_3_O_5_]^+^, [ArB_4_O_6_]^+^ and [ArB_5_O_7_]^+^, respectively, at the CCSD(t)/cc-pVTZ level, which are larger than that of the well-studied [CH_3_Ar]^+^ complex.^48^ The Ar–B bond distance, Wiberg bond order and bond dissociation energy of the [ArB_3_O_4_]^+^ cation complex were predicted to be 2.851 Å, 0.015 and 3.4 kcal mol^–1^, indicating that the Ar–B interaction in [ArB_3_O_4_]^+^ is much weaker than those in the Ar–boroxol ring cation complexes.

**Table 2:** Energy decomposition analyses of [ArB_3_O_4_]^+^, [ArB_3_O_5_]^+^, [ArB_4_O_6_]^+^ and [ArB_5_O_7_]^+^ at the BP86/TZ2P level using the CCSD(T)/cc-pVTZ optimized geometries. The Ar–B bond distances (Å), dissociation energies (*D*_e_), natural charges (*Q*) and Wiberg bond orders (WBI) are also shown. Energy values are given in kcal mol^–1^

Species	Ar–B_3_O_4_^+^(^1^A_1_)	Ar–B_3_O_5_^+^(^3^B_2_)	Ar–B_4_O_6_^+^(^2^A′)	Ar–B_5_O_7_^+^(^1^A_1_)
*r*(Ar–B)	2.851	2.004	2.002	2.001
*D*_e_	3.4	16.2	16.3	16.4
Δ*E*_int_	–2.3	–19.7	–19.5	–20.4
Δ*E*_Pauli_	6.2	53.1	54.1	51.2
Δ*E*_elstat_ ^*a*^	–1.6[18.8%]	–15.0[20.7%]	–16.1[21.9%]	–14.3[20.0%]
Δ*E*_orb_ ^*a*^	–6.9[81.2%]	–57.8[79.3%]	–57.5[78.1%]	–57.3[80.0%]
Δ*E*_orb_ σ^*b*^	–5.1(73.9%)	–44.7(77.3%)	–45.0(78.3%)	–45.3(79.1%)
Δ*E*_orb_ π^*b*^		–8.6(14.9%)	–9.2(16.0%)	–9.0(15.7%)
Δ*E*_orb(r)_ ^*b*^	–1.8(26.1%)	–4.5(7.8%)	–3.3(5.7%)	–3.0(5.2%)
*Q*(Ar)	0.036	0.355	0.357	0.358
WBI	0.015	0.543	0.545	0.547

^*a*^The values in brackets give the percentage contribution to the total attractive interactions Δ*E*_elstat_ + Δ*E*_orb_.

^*b*^The values in parentheses give the percentage contribution to the total Δ*E*_orb_. Δ*E*_orb(r)_ is the rest interaction energy of Δ*E*_orb_.

**Fig. 4:**
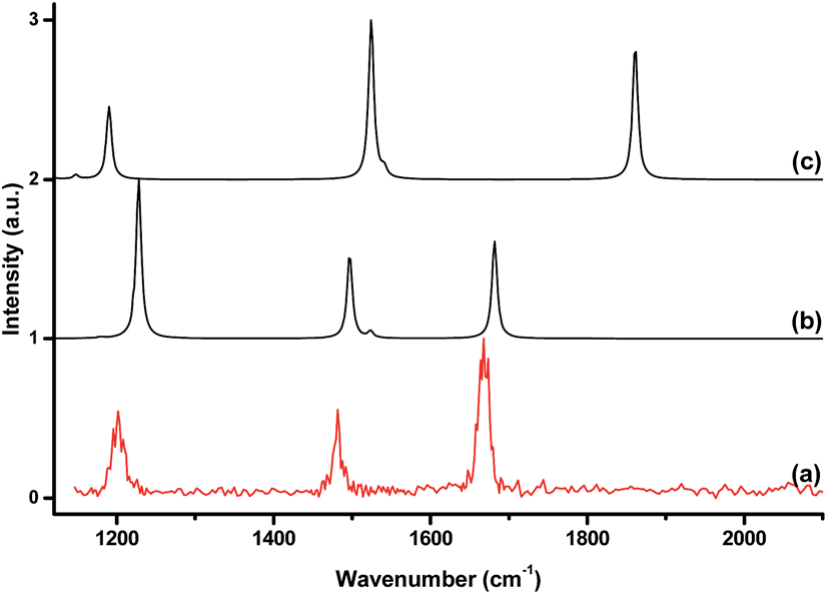
The experimental IR spectrum of [ArB_3_O_5_]^+^ (a) and the simulated IR spectra of [ArB_3_O_5_]^+^ (b) and B_3_O_5_^+^ (c) at the B3LYP-D3/aug-cc-pVTZ level.

Consistent with the large bond dissociation energies, the binding of the argon atom had a strong influence on the geometries of the B_3_O_5_^+^, B_4_O_6_^+^ and B_5_O_7_^+^ cation clusters. Large changes in the bond angles and lengths were observed in the boroxol ring upon argon bonding (Fig. 3 and S5†). The O–B–O angle of the argon coordinated OBO moiety was more acute and the two B–O bonds were slightly longer, while the two adjacent O–B bonds were shorter than those of the bare B_3_O_5_^+^, B_4_O_6_^+^ and B_5_O_7_^+^ cation clusters. Fig. 4 shows a comparison between the experimental IR spectrum of [ArB_3_O_5_]^+^ and the simulated IR spectra with and without argon bonding. It clearly shows that the argon bonding had a significant effect on the IR spectrum of [B_3_O_5_]^+^, particularly on the antisymmetric stretching mode of the argon-coordinated OBO moiety, which was red-shifted by about 180 cm^–1^ upon argon bonding.

**Fig. 5:**
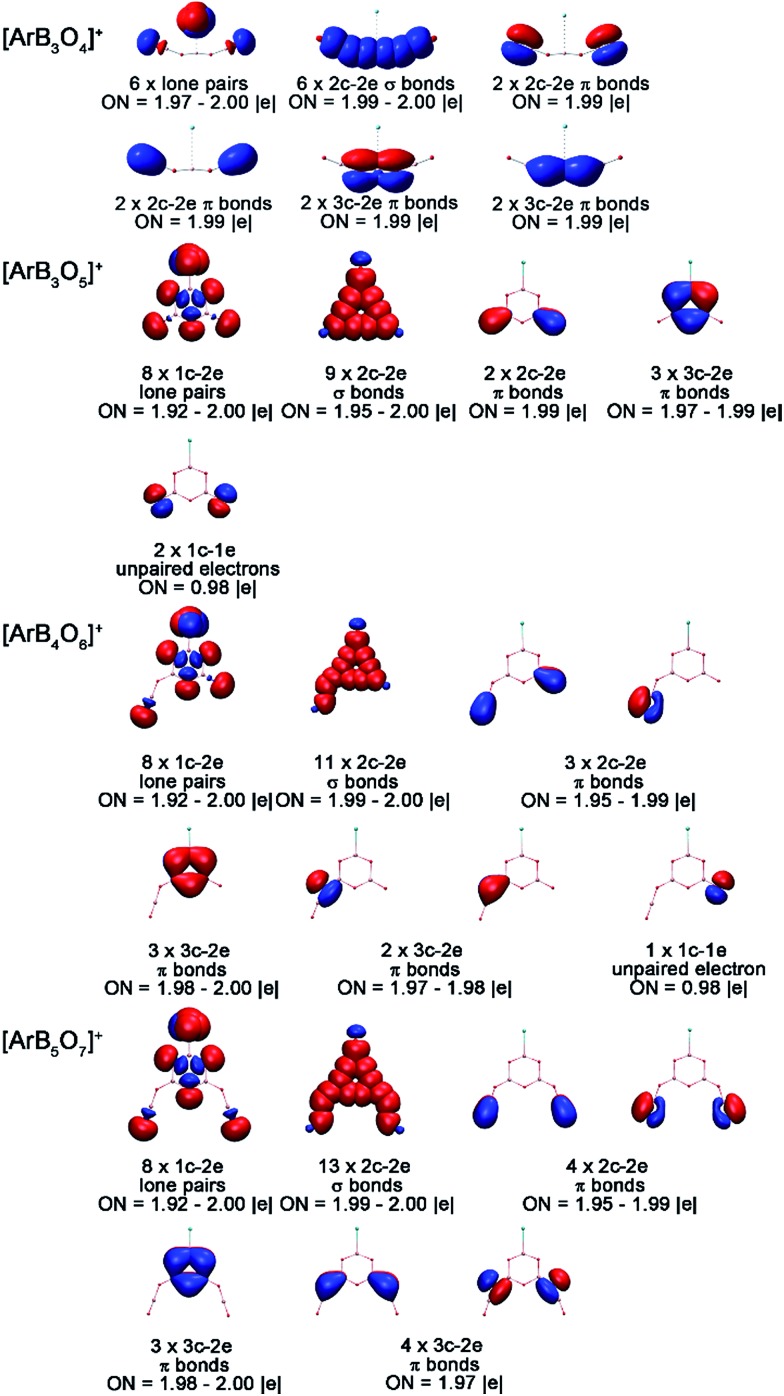
AdNDP bonding orbitals of the [ArB_3_O_4_]^+^, [ArB_3_O_5_]^+^, [ArB_4_O_6_]^+^ and [ArB_5_O_7_]^+^ cation complexes. ON stands for occupation number.

To understand the bonding in these cation complexes, AdNDP analysis was carried out (Fig. 5). This method has been successfully used to analyze chemical bonding in boron clusters, aromatic molecules and gold clusters.^49^ The analyses of [ArB_3_O_5_]^+^, [ArB_4_O_6_]^+^ and [ArB_5_O_7_]^+^ revealed one 2c–2e σ bond with an occupation number (ON) of 1.99 for each cation complex, which was formed between the in-plane 2p atomic orbitals of the argon and boron. No such Ar–B bond can be found in the [ArB_3_O_4_]^+^ cation complex with the chain structure. For each boroxol ring cation complex, there are three 3c–2e delocalized π bonds within the boroxol ring, satisfying the 4*n* + 2 rule for aromaticity, similar to the B_4_O_4_^+^ cations reported previously.^50^ Thus, all three boroxol ring cation clusters are π aromatic.

**Fig. 6:**
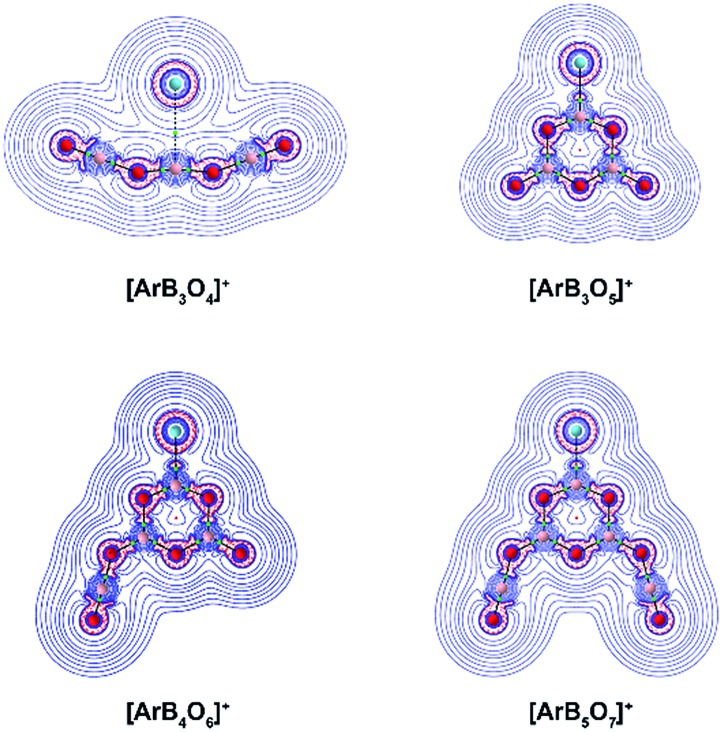
Contour diagrams of the Laplacian of the electron density (∇^2^ *ρ*(*r*)) of [ArB_3_O_4_]^+^, [ArB_3_O_5_]^+^, [ArB_4_O_6_]^+^ and [ArB_5_O_7_]^+^ in the molecular plane. The black sticks represent the bond path (BP), and tiny green points illustrate the bond critical point (BCP). The dashed red lines indicate areas of charge concentration (∇^2^ *ρ*(*r*) < 0) while solid blue lines show areas of charge depletion (∇^2^ *ρ*(*r*) > 0).

We investigated the topology of the electron density distribution of the species using the quantum theory of atoms in molecules (QTAIM) method.^35^ Fig. 6 shows the Laplacian of the electron density (∇^2^ *ρ*(*r*)) of the cation complexes in the molecular plane (the side view is shown in Fig. S11†). There is a bond critical point (∇*ρ*(*r*) = 0 and ∇^2^ *ρ*(*r*) < 0 in one direction) and an associated bond path for the Ar–B moiety in each cation complex. The Laplacian electron density distribution clearly shows a depletion region (∇^2^ *ρ*(*r*) > 0) between argon and boron in [ArB_3_O_4_]^+^ and charge concentration “islands” (∇^2^ *ρ*(*r*) < 0, red dashed lines) between boron and argon in the boroxol ring complexes, suggesting a noncovalent Ar–B interaction in [ArB_3_O_4_]^+^ and a genuine Ar–B dative bond in the boroxol ring cation complexes. Natural bond orbital analysis indicated that the argon atom carries positive charges of around 0.36*e* in the argon–boroxol ring species, but positive charges of only 0.04*e* in [ArB_3_O_4_]^+^ (Table 2).

**Fig. 7:**
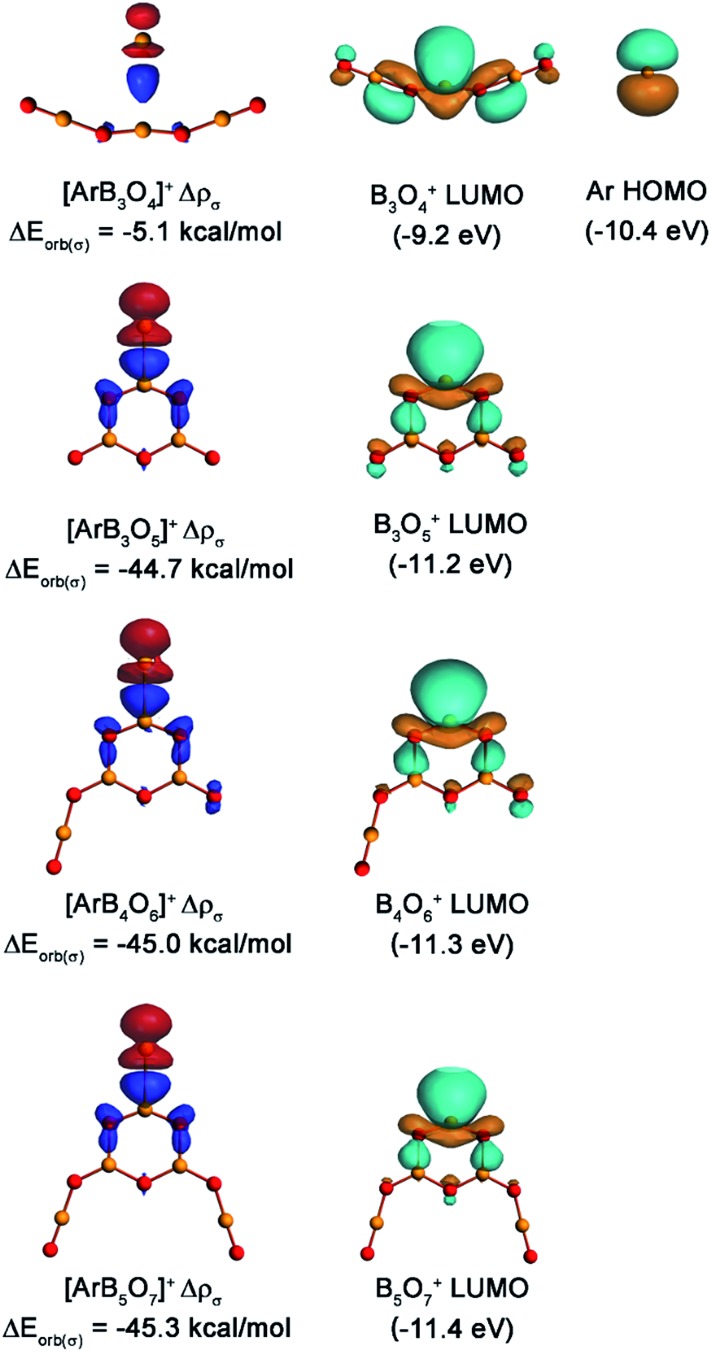
Plots of the deformation densities Δ*ρ* (isocontour 0.002 a.u.) of the pairwise σ orbital interactions between the argon and boron oxide cations in the [ArB_3_O_4_]^+^, [ArB_3_O_5_]^+^, [ArB_4_O_6_]^+^ and [ArB_5_O_7_]^+^ cations and the pairwise interaction orbitals, the LUMO of bare cations and HOMO of the argon atom. The orbital interaction energies and the fragments’ orbital energies were calculated at the BP86/TZ2P level. The charge flow of the σ orbital interactions is red to blue.

The nature of the Ar–B interactions was further analyzed with the EDA-NOCV method,^36^ which gave detailed insight into the bonding situation. The numerical results of the Ar–B interactions are listed in Table 2. It is quite obvious that the orbital interactions came mainly from the σ donation of argon to the positively charged boron center, which contributes 73.9% of the orbital energy (Δ*E*_orb_) in [ArB_3_O_4_]^+^, 77.3% in [ArB_3_O_5_]^+^, 78.3% in [ArB_4_O_6_]^+^, and 79.1% in [ArB_5_O_7_]^+^. The contribution of the π donation was much weaker than the σ donation. Fig. 7 displays the deformation densities Δ*ρ*(σ) which are connected to the σ donation interactions (the plots of deformation densities of π orbital interaction are given in Fig. S12†). The color code for the charge flow is red to blue. The shape of the deformation density for [ArB_3_O_4_]^+^ implies that the orbital interaction only caused the charge transfer from argon to the region between argon and boron. This kind of interaction can be considered as an ion-induced weak dipole interaction, as reported previously.^18*a*^ The shapes of the deformation densities for the [ArB_3_O_5_]^+^, [ArB_4_O_6_]^+^ and [ArB_5_O_7_]^+^ cations indicate that the charge flow comes mainly from the in-plane lone-pair electrons at argon to the BO_2_ moiety of the boroxol ring, which leads to charge accumulation at the boron atom and the two adjacent O–B bonds (blue region). Note that the interaction energies (Δ*E*_int_) and the orbital energies (Δ*E*_orb_) of the [ArB_3_O_5_]^+^, [ArB_4_O_6_]^+^ and [ArB_5_O_7_]^+^ cation complexes were smaller than those of the strongly covalent H–Ar bond in HArF, but were much larger than those of the [ArB]^+^ cation, which has been characterized as involving weak bonding interaction with some covalent or electrostatic properties.^18*a*^ Fig. 7 also displays the pairwise interaction orbitals, the lowest unoccupied molecular orbitals (LUMOs) of bare cations and the highest occupied molecular orbital (HOMO) of argon. Although the LUMO of B_3_O_4_^+^ exhibits very similar spatial distribution to those of the boroxol ring cations, it lies 1.2 and about 2.0 eV higher in energy than the HOMO of argon (–10.4 eV) and the LUMO of the boroxol ring cations (–11.2 to –11.4 eV), respectively. This leads to a much weaker donation interaction in [ArB_3_O_4_]^+^ than those in [ArB_3_O_5_]^+^, [ArB_4_O_6_]^+^, and [ArB_5_O_7_]^+^.

## Conclusions

Boron oxide–argon cation complexes in the form of [ArB_3_O_4_]^+^, [ArB_3_O_5_]^+^, [ArB_4_O_6_]^+^ and [ArB_5_O_7_]^+^ were prepared in the gas phase and were mass-selected and studied by infrared photodissociation spectroscopy. Spectroscopy combined with quantum chemical calculations revealed that the [ArB_3_O_4_]^+^ cation is a weakly bound Ar-tagged complex with a B_3_O_4_^+^ chain structure. In contrast, the [ArB_3_O_5_]^+^, [ArB_4_O_6_]^+^ and [ArB_5_O_7_]^+^ cations all have planar structures each involving an aromatic boroxol ring and an argon–boron covalent bond. The [ArB_3_O_5_]^+^, [ArB_4_O_6_]^+^ and [ArB_5_O_7_]^+^ cations reported here represent the very first examples of spectroscopically characterized complexes featuring strong dative bonding between argon and boron that are stable in the gas phase.
