# Supplementary material for: Preparation and characterization of chemically bonded argon–boroxol ring cation complexes
Source: Chem Sci. 2017 Jul 17;8(9):6594–600. doi: 10.1039/c7sc02472j (PMC5627188; doi:10.1039/c7sc02472j)
Supplement: Supplementary file 1 [file SC-008-C7SC02472J-s001.pdf]

Electronic Supplementary Material (ESI) for Chemical Science.
This journal is © The Royal Society of Chemistry 2017

Supplementary Materials

Preparation and Characterization of Chemically Bonded Argon-Boroxol Ring Cation Complexes

Jiaye Jin, Wei Li, Yuhong Liu, Guanjun Wang, Mingfei Zhou*

Department of Chemistry, Collaborative Innovation Center of Chemistry for Energy Materials, Shanghai Key Laboratory of Molecular Catalysis and Innovative Materials, Fudan University, Shanghai 200433, China, E-mail: mfzhou@fudan.edu.cn

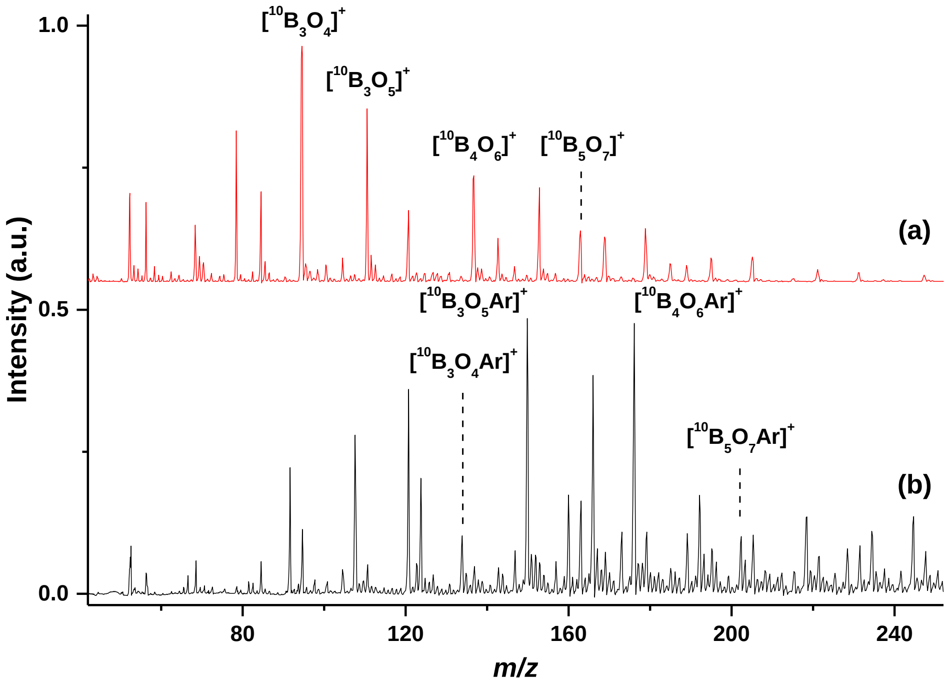

Figure S1. Mass spectra of boron oxide cation clusters in the m/z range of 40-250 produced by pulsed laser evaporation of a ^{10}B-enriched boron target in expansion of (a) helium seeded with 1% O_2 (red line), and (b) helium seeded with 10% argon and 1% O_2 (black line).

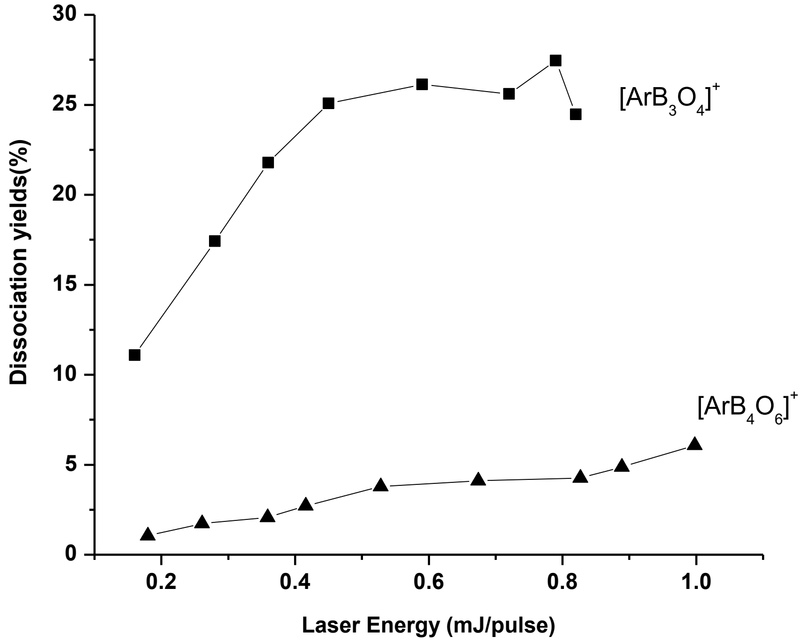

Figure S2. Plots of the photodisssociation yields of $[ArB_3O_4]^+$ and $[ArB_4O_6]^+$ as a function of IR laser energy.

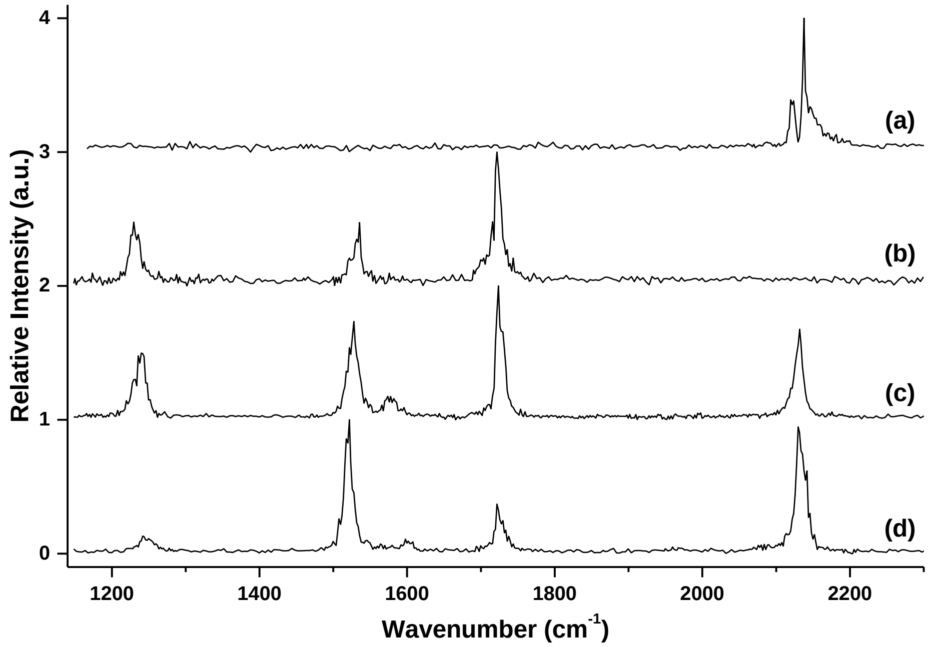

Figure S3. Infrared photodissociation spectra of mass-selected cation complexes in the 1150-2300 cm^{-1} region measured by monitoring the argon photodissociation channel. (a) $[Ar^{10}B_3O_4]^+$, (b) $[Ar^{10}B_3O_5]^+$, (c) $[Ar^{10}B_4O_6]^+$, and (d) $[Ar^{10}B_5O_7]^+$.

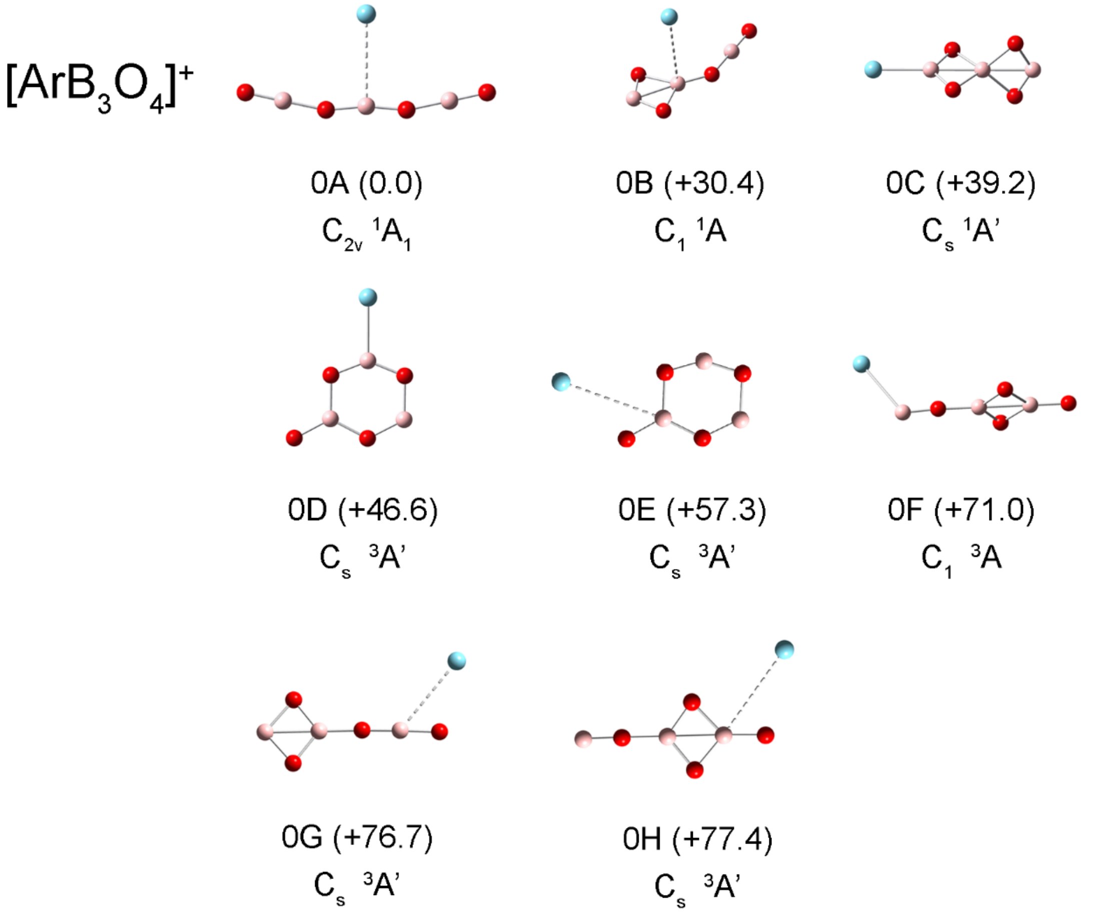

Figure S4. Optimized structures of the $[ArB_3O_4]^+$ isomers at the B3LYP / aug-cc-pVTZ level. The symmetry, electronic states, and relative energies with zero-point energy corrected (kcal/mol) are shown in the figure.

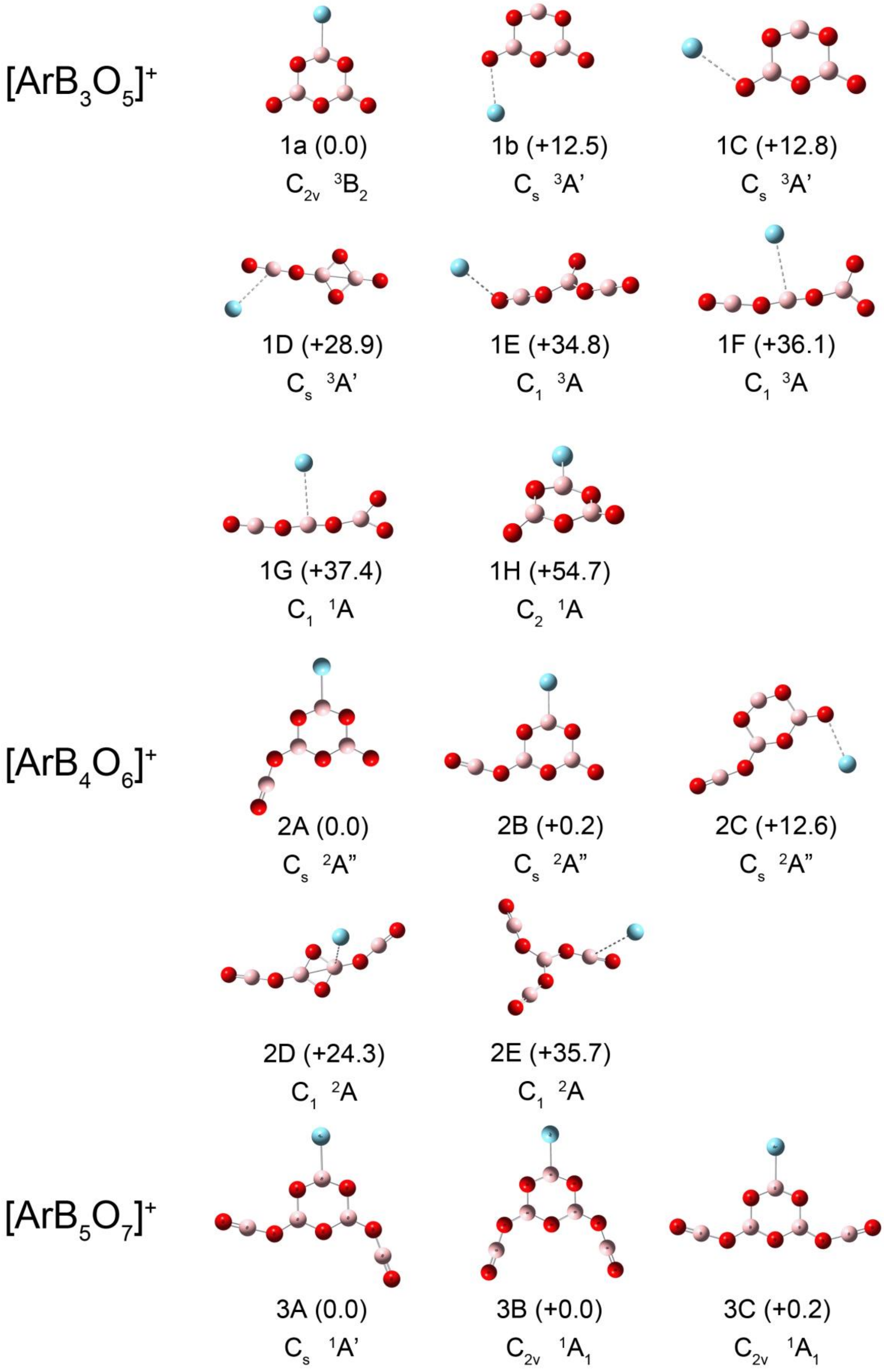

Figure S5. Optimized structures of the $[ArB_3O_5]^+$, $[ArB_4O_6]^+$, and $[ArB_5O_7]^+$ isomers at the B3LYP / aug-cc-pVTZ level. The symmetry, electronic states, and relative energies with zero-point energy corrected (kcal/mol) are shown in the figure.

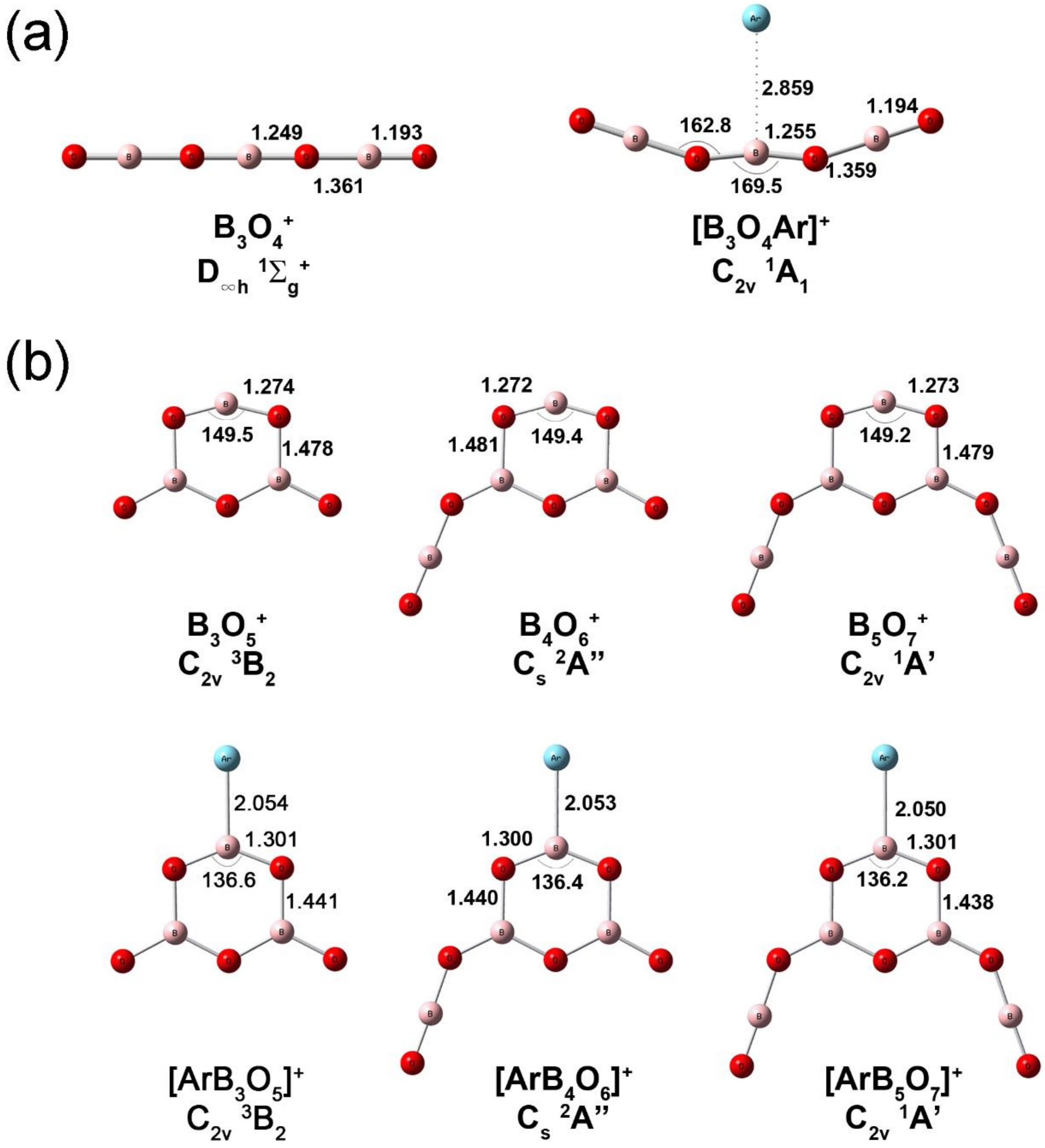

Figure S6. Optimized structures of the most stable structure of the $[ArB_3O_4]^+$, $[ArB_3O_5]^+$, $[ArB_4O_6]^+$ and $[ArB_5O_7]^+$ cation complexes and the bare $[B_3O_4]^+$, $[B_3O_5]^+$, $[B_4O_6]^+$ and $[B_5O_7]^+$ cations at the B3LYP-D3/aug-cc-pVTZ level of theory. The bond lengths are given in angstroms and bond angles in degrees.

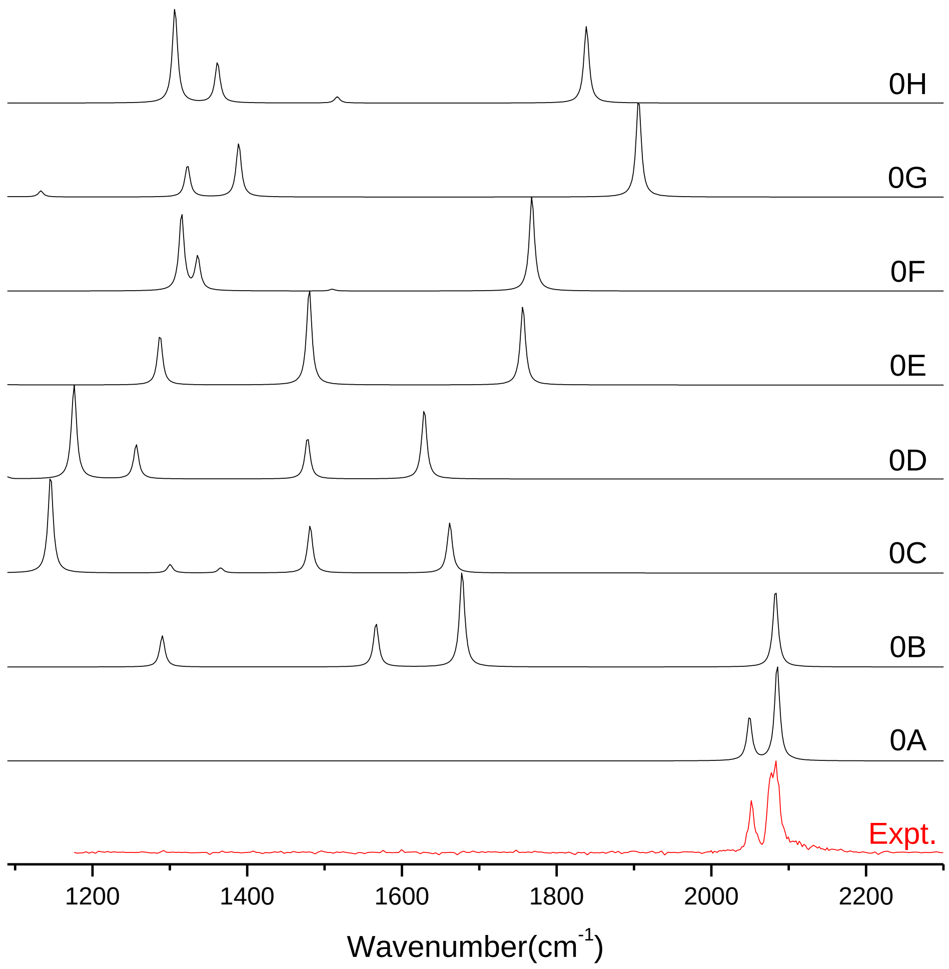

Figure S7. Experimental (red) and simulated vibrational spectra of $[Ar^{11}B_3O_4]^+$ in the 1090-2300 cm^{-1} region. The simulated spectra are obtained from unscaled harmonic vibrational frequencies and intensities calculated at the B3LYP-D3/aug-cc-pVTZ level for the structures shown in Figure S3.

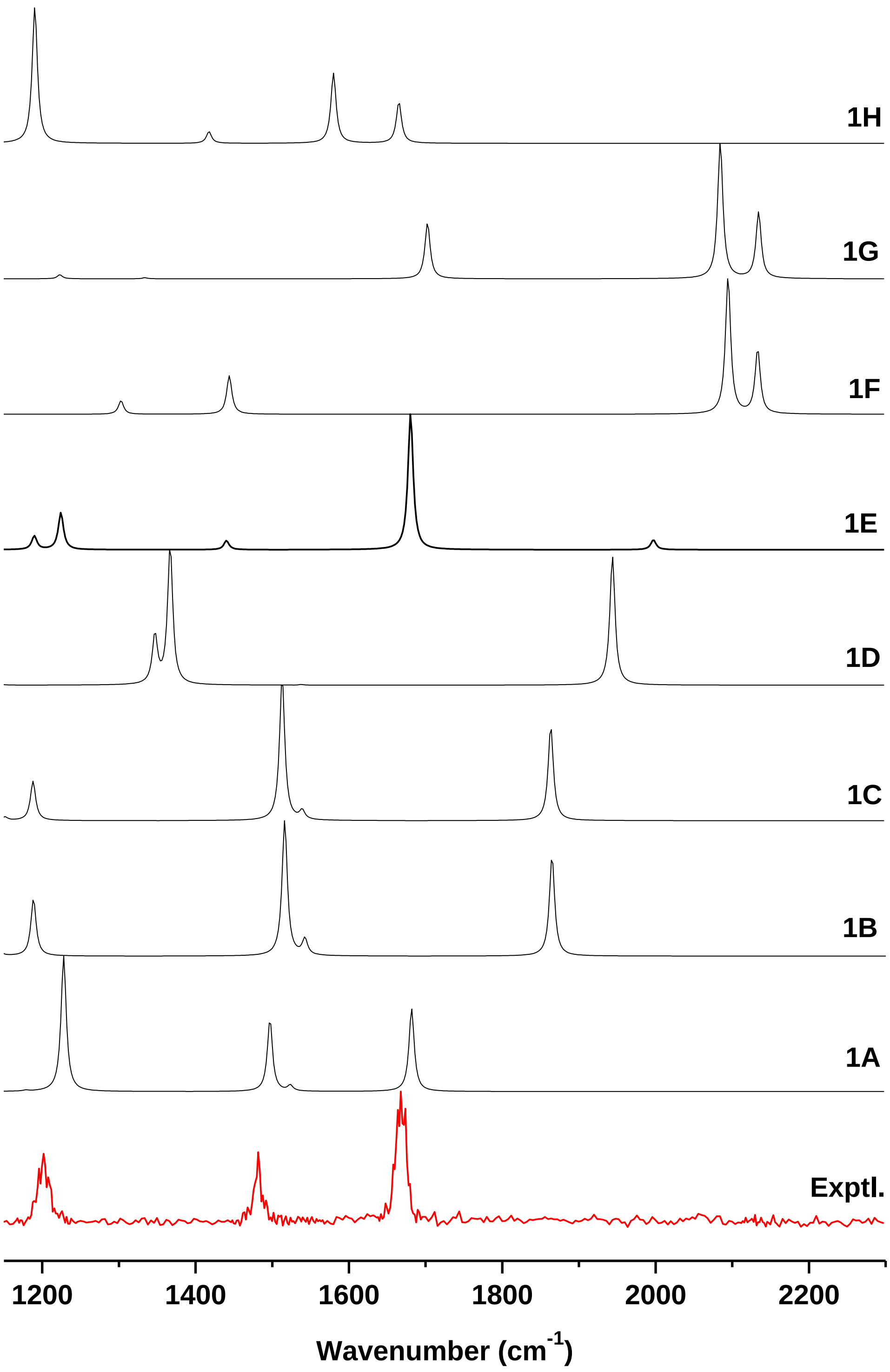

Figure S8. Experimental (red) and simulated vibrational spectra of $[Ar^{11}B_3O_5]^+$ in the 1150-2300 cm^{-1} region. The simulated spectra are obtained from unscaled harmonic vibrational frequencies and intensities calculated at the B3LYP-D3/aug-cc-pVTZ level for the structures shown in Figure S4.

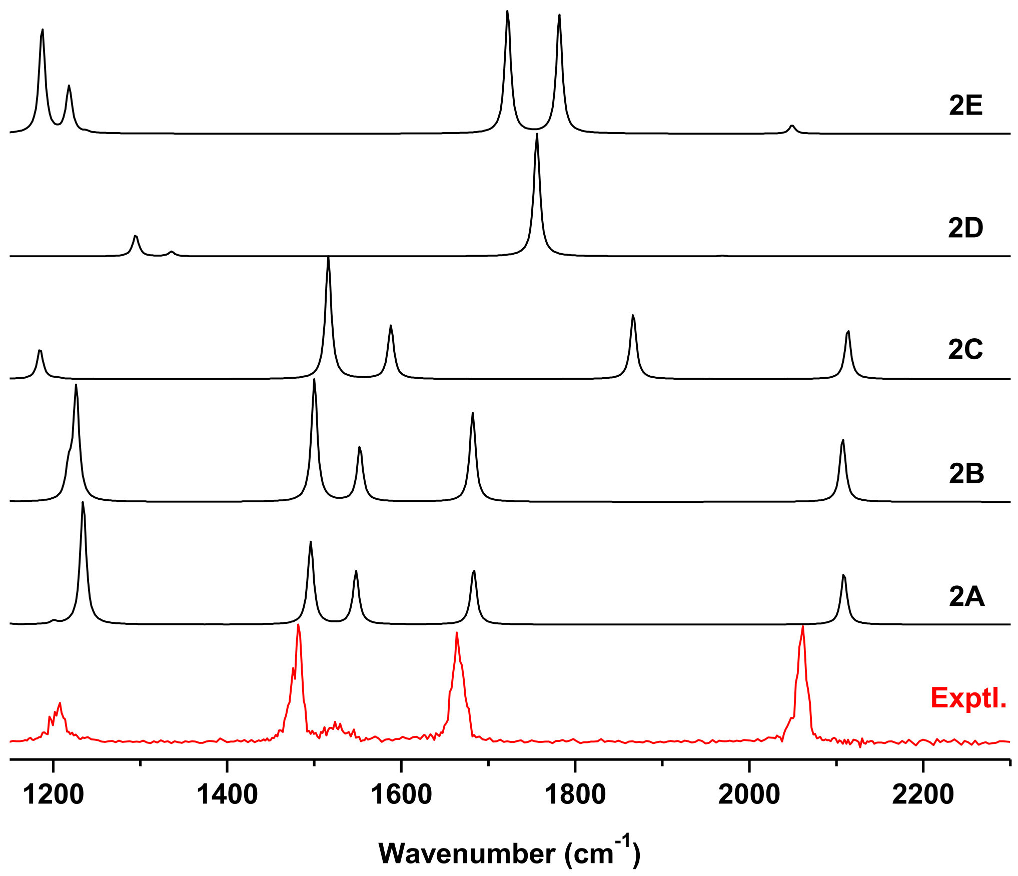

Figure S9. Experimental (red) and simulated vibrational spectra of $[Ar^{11}B_4O_6]^+$ in the 1150-2300 cm^{-1} region. The simulated spectra are obtained from unscaled harmonic vibrational frequencies and intensities calculated at the B3LYP-D3/aug-cc-pVTZ level for the structures shown in Figure S4.

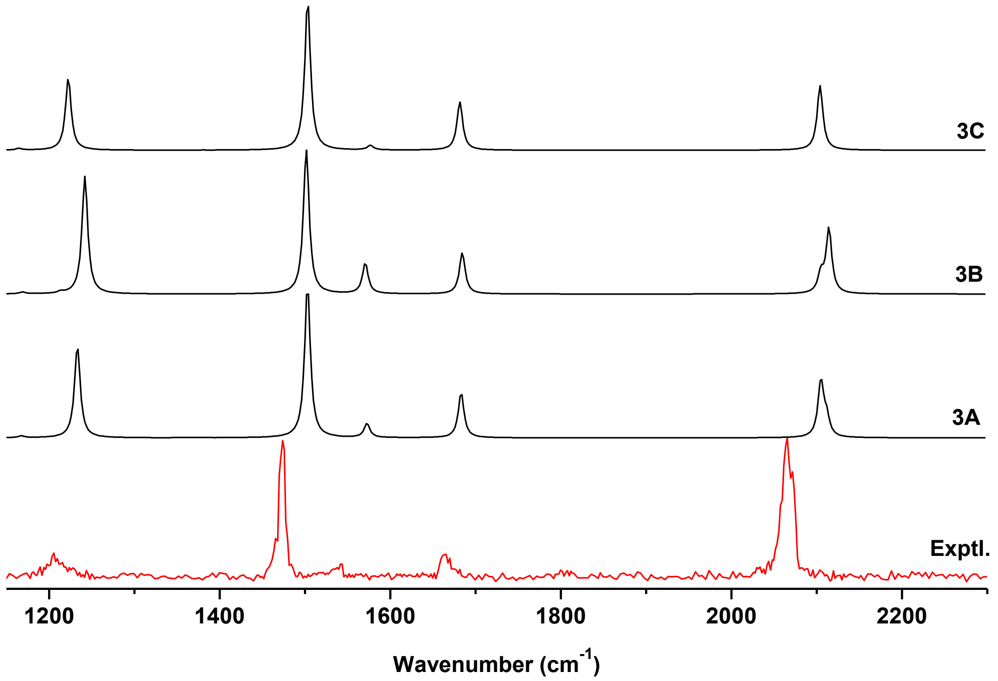

Figure S10. Experimental (red) and simulated vibrational spectra of $[Ar^{11}B_5O_7]^+$ in the 1150-2300 cm^{-1} region. The simulated spectra are obtained from unscaled harmonic vibrational frequencies and intensities calculated at the B3LYP-D3/aug-cc-pVTZ level for the structures shown in Figure S4.

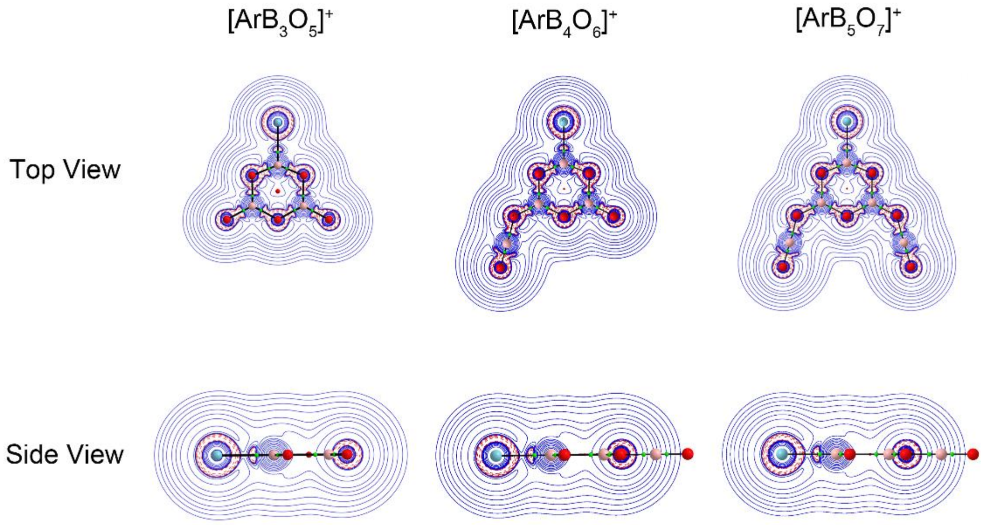

Figure S11. Contour diagrams of the Laplacian of the electron density ($\nabla^2\rho(r)$) of $[ArB_3O_5]^+$, $[ArB_4O_6]^+$ and $[ArB_5O_7]^+$ in the top view (molecular plane) and side view.

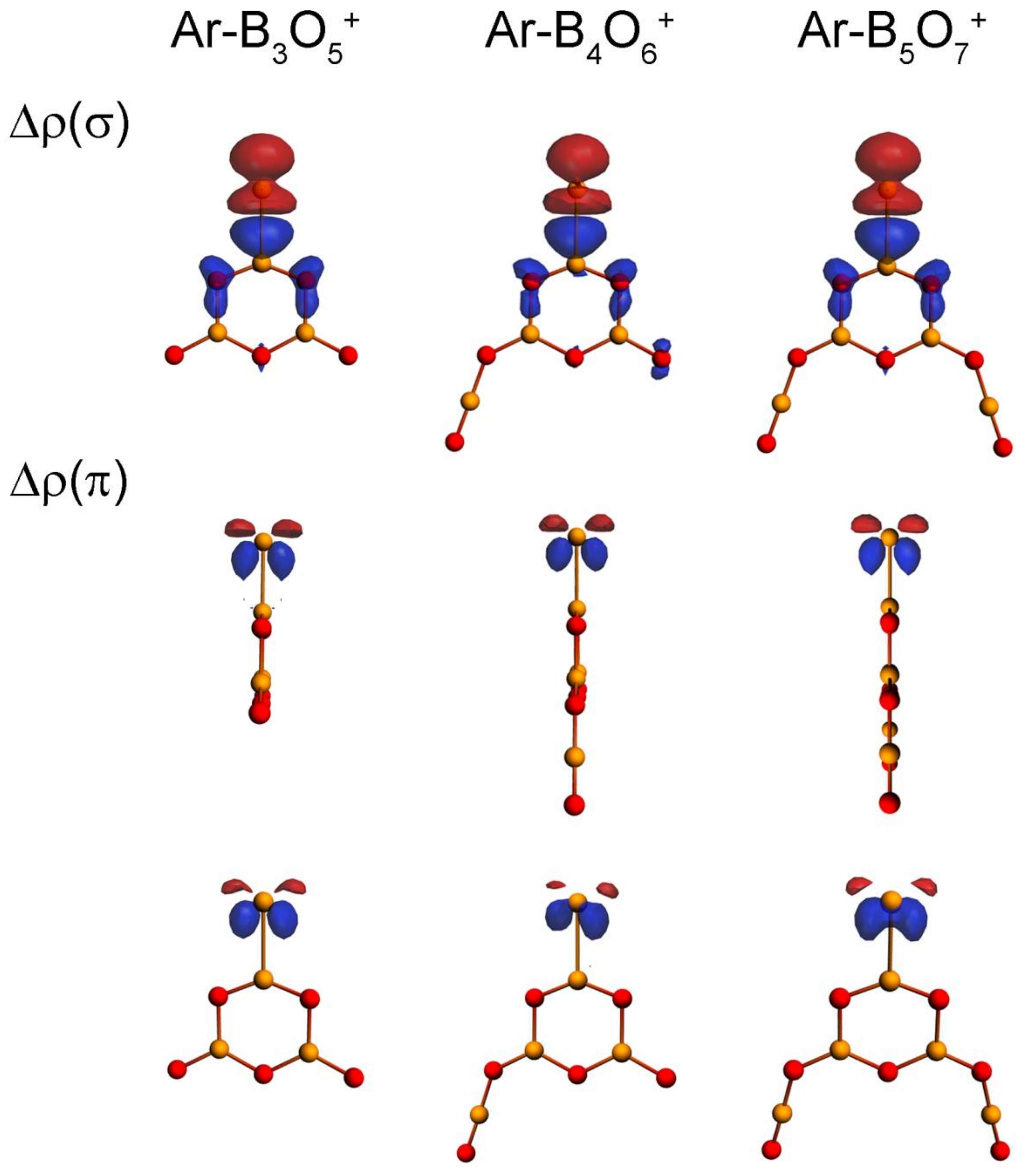

Figure S12. Plots of deformation densities Δρ of the pairwise σ and π orbital interactions between Ar and boron oxide cation fragments in $[ArB_3O_5]^+$, $[ArB_4O_6]^+$, and $[ArB_5O_7]^+$. The direction of charge flow is red to blue.
